# Modulation of p-eIF2α cellular levels and stress granule assembly/disassembly by trehalose

**DOI:** 10.1038/srep44088

**Published:** 2017-03-09

**Authors:** Pasquale Dimasi, Annamaria Quintiero, Tatyana A. Shelkovnikova, Vladimir L. Buchman

**Affiliations:** 1 School of Biosciences, Cardiff University, Museum Avenue, Cardiff, CF10 3AX, United Kingdom; 2 Institute of Physiologically Active Compounds Russian Academy of Sciences, 1 Severniy proezd, Chernogolovka 142432, Moscow Region, Russian Federation

## Abstract

Stress granules (SGs) are an important component of cellular stress response. Compromised assembly of SGs as well as their premature or delayed disassembly affect physiology and survival of cells under stress or during recovery from stress. Consequently, abnormal turnover of SGs has been implicated in the development of various pathologies, including neurodegeneration. We found that pretreatment of cells with a natural disaccharide trehalose, a known autophagy enhancer, delays SG assembly and facilitates their premature post-stress disassembly. Mechanistically, the effect of trehalose on SGs is mediated via the p-eIF2α rather than autophagosome pathway. Trehalose increases pre-stress levels of p-eIF2α and its phosphatase subunits and promotes post-stress translational recovery. Upon prolonged treatment, trehalose impairs basal translation affecting production of transiently expressed proteins. Early translational recovery and SG disassembly induced by trehalose pretreatment can sensitise cells to stress and impair survival. Our study has important implications for the use of trehalose in studies of autophagic clearance of misfolded proteins and for targeting SGs as a possible therapeutic approach in neurodegenerative and other diseases.

Stress granules (SGs) are cytoplasmic RNA-protein complexes found in the majority of cultured eukaryotic cells, from yeast to human, under specific stress conditions^1^. SGs comprise a variety of proteins some of which are essential for SG assembly and others are recruited in a piggy-back fashion. The majority of SG proteins contain either a low-complexity (prion-like) domain, which facilitates phase transition, or a RNA-binding domain(s), which allows interaction with core SG components, or both^2^. Among functions of SGs are protection of cellular RNAs from degradation; mRNA triage during stress; participation in stress signaling; protection from cell death^3^,^4^,^5^,^6^,^7^,^8^. For most stresses, SG formation is initiated by increased phosphorylation of the α subunit of eukaryotic initiation factor 2 (eIF2), a component of the eIF2-GTP-tRNAiMet ternary complex^9^. This event blocks polysome assembly and halts translation. However, in some cases SGs are formed independent of eIF2α phosphorylation and can be triggered by inhibition of other translation initiation factors such as eIF4A^10^.

Many SG proteins are known to be dysregulated in human disease, first of all, in amyotrophic lateral sclerosis (ALS)^11^,^12^,^13^. Some ALS-associated SG proteins have been reported to regulate SG assembly and dynamics^14^,^15^,^16^,^17^,^18^,^19^. Enrichment of SGs with proteins containing low-complexity domains suggested that they can become ‘seeds’ for subsequent secondary aggregation of these proteins and development of a proteinopathy. In this scenario, SGs or SG-like assemblies containing abnormal proteins might persist in cells longer than required for normal SG-associated signaling giving rise to proteinaceous inclusions^12^,^20^. Therefore, approaches aimed at promoting timely disassembly of SGs are considered as means to prevent accumulation and aggregation of specific proteins. A class of such compounds recently reported to promote SG disassembly are autophagy enhancers^21^.

In present study we tested a panel of established autophagy enhancers with different mechanism of action. To our surprise, the only compound capable of promoting SG disassembly was a disaccharide trehalose. We further established that the effect of trehalose on SGs is mediated primarily via the p-eIF2α pathway.

## Results

### Trehalose pretreatment facilitates stress granule disassembly

**Figure 1:**
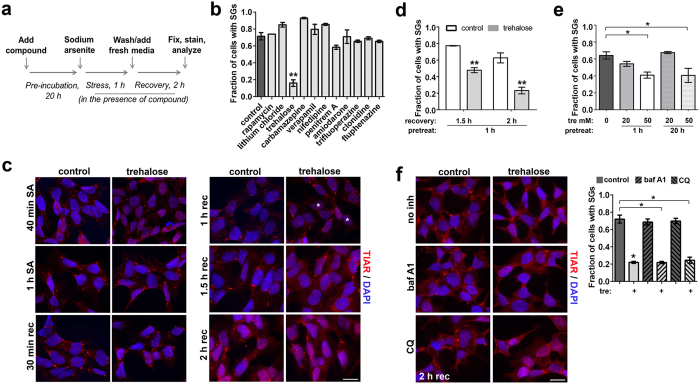
Trehalose facilitates the disassembly of sodium arsenite-induced stress granules after stress in autophagy-independent manner. (**a**) Experimental setup to identify compounds accelerating SG disassembly after stress. (**b**) Among tested autophagy-enhancing compounds, trehalose at 100 mM was the only one that significantly accelerated SG disassembly during recovery from oxidative stress (n = 4, **p < 0.01). (**c**) The dynamics of SG disassembly in control and trehalose-treated cultures. The cells were pretreated with trehalose (100 mM) for 20 h and fixed at the indicated time-points during the recovery and stained with an antibody against a core SG protein TIAR. Representative images for each time point are shown; asterisks indicate cells free from SGs. Scale bar, 10 μm. (**d**) Short (1 h) pretreatment with trehalose (100 mM) is sufficient to decrease the fraction of cells with SGs during the recovery from oxidative stress (n = 3, **p < 0.01). (**e**) The effect of trehalose on SGs is concentration-dependent. Cells were pretreated with the indicated trehalose concentrations for 1 h or 20 h and analysed after 2 h of recovery (n = 3, *p < 0.05). (**f**) Inhibition of autophagy does not affect the ability of trehalose to facilitate SG disassembly. The cells were pretreated with trehalose (100 mM) and autophagy inhibitors bafilomycin A1 (baf A1) or chloroquine (CQ) for 1 h, stressed with SA and left to recover for 2 h in the presence of trehalose and/or inhibitors. Representative images and quantitation for each condition are shown. N = 3, *p < 0.05. Scale bar, 10 μm.

A recent study^21^ suggested that autophagy plays a role in the clearance of SGs. Therefore our initial aim was to test known autophagy enhancers for their ability to accelerate SG disassembly and identify the most efficient compounds. A simple quantitative approach was utilised: neuroblastoma SH-SY5Y cells were pretreated with each compound for 20 h; subjected to a reversible oxidative stress (sodium arsenite, SA) for 1 h in the presence of the compound; washed from SA and left to recover for 2 h in the presence of the compound; stained for an established SG marker TIAR (Fig. 1a). The fraction of cells with at least two large (mature) SGs was determined for each compound. In this experimental setting, ~30% of control cells clear SGs from cytoplasm after 2 h of recovery. Unexpectedly, among autophagy-inducing compounds with distinct mechanisms of action, a significant decline of the number of cells with SGs was detected only in trehalose-treated cultures (Fig. 1a,b). This effect was due to accelerated SG disassembly since virtually all cells developed two and usually more SGs in the presence of trehalose after 1 h of SA treatment (Fig. 1c).

Consistent with the previous report^22^, we observed visible conversion of LC3I to LC3II, a marker of activated autophagy, only after prolonged, 16–24 h, exposure to 100 mM trehalose (Supplementary Fig. 1a,b). However, not only such long but also significantly shorter pretreatment (1 h) or lower concentration (50 mM) of trehalose that did not induce detectable LC3 conversion promoted SG disassembly during recovery from SA (Fig. 1d,e). To assess whether autophagy-stimulating properties of trehalose contribute to its ability to promote SG clearance, we used two late-stage autophagy inhibitors, bafilomycin A1 and chloroquine. As expected, both compounds cause increased levels of LC3B-II isoform after 3 h of treatment indicating accumulation of defective autophagosomes (Supplementary Fig. 1c). However, co-treatment with these inhibitors did not alter the ability of trehalose to promote SG disassembly post-stress (Fig. 1f).

Thus trehalose facilitates SG disassembly via an autophagy-independent route.

### Trehalose promotes post-stress p-eIF2α dephosphorylation and translational recovery

**Figure 2:**
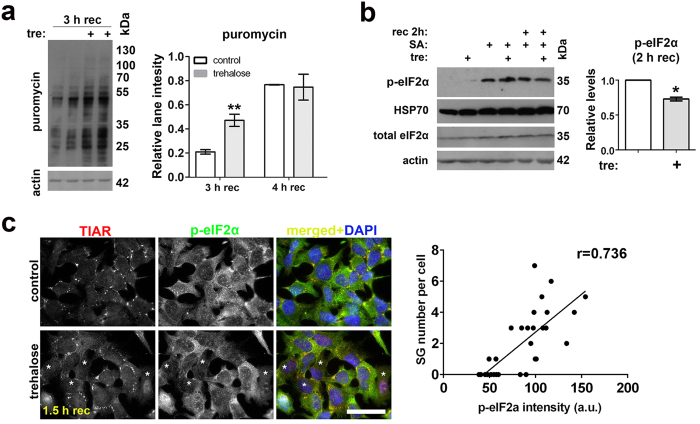
Trehalose decreases post-stress p-eIF2α levels and promotes translational recovery. (**a**) Trehalose accelerates translational recovery after stress. Cells were pretreated with trehalose (100 mM) for 1 h, stressed with SA for 1 h, and after washing off SA left to recover for 3 or 4 h. Newly synthesised proteins were labeled with puromycin for 30 min and detected by Western blotting. A representative blot and quantification of lane intensities are shown (n = 4, **p < 0.01). (**b**) During the recovery from SA stress, p-eIF2α is dephosphorylated more rapidly in cells pretreated with trehalose (100 mM for 1 h). Antibody recognising eIF2α phosphorylated at Ser51 were used for Western blot; a representative blot and quantification of p-eIF2α band intensities for the 2 h recovery time-point are shown (n = 3, *p < 0.05). Full-size images of blots are available in the Supplementary Information. (**c**) Immunocytochemistry revealed that cells with diminished p-eIF2α signal in the cytoplasm are often free from SGs (labeled with asterisks). Correlation analysis demonstrated that decrease in fluorescence intensity of p-eIF2α in the cytoplasm correlates with SG disassembly during recovery from stress (p < 0.0001). Cells were analysed after 1.5 h of recovery from SA; a.u. – arbitrary units. Scale bar, 10 μm.

Since SG disassembly is usually coupled with restoration of translation, we measured the effect of trehalose pretreatment on translation after stress. We pretreated cells with 100 mM trehalose for 1 h, stressed cells with SA for 1 h and assessed puromycin incorporation by Western blot during recovery from this stress. Puromycin signal appeared after 3 h of recovery, and at this time point we detected significantly higher translational rates in trehalose-pretreated cells whereas the difference was no longer detectable after 4 h of recovery (Fig. 2a). This indicated that trehalose pretreatment accelerates restoration of translation.

SG disassembly is linked to dephosphorylation of p-eIF2α, which promotes translational recovery^9^. We therefore assessed the effect of trehalose pretreatment on levels of p-eIF2α (Ser51) in cells either only treated for 2 h; or pretreated with trehalose for 1 h and stressed with SA for 1 h; or pretreated with trehalose for 1 h, stressed with SA for 1 h and recovered for 2 h. Approximately 25% decrease in p-eIF2α levels was detected in trehalose-pretreated cells after 2 h of recovery compared to control cells (Fig. 2b), and there was a correlation between cellular p-eIF2α levels and the number of SGs per cell (Fig. 2c). At the same time, the level of HSP70 chaperone which is known to facilitate SG disassembly^23^ was not altered in response to trehalose treatment (Fig. 2b).

Similar decrease in post-stress p-eIF2α levels was observed for the 24 h trehalose pretreatment (Supplementary Fig. 1d) and without continuous presence of trehalose in the media (i.e. 1 h pretreatment only) (Supplementary Fig. 1e). In sharp contrast, lack of pretreatment (trehalose addition together with SA or immediately before recovery) increased p-eIF2α levels and did not have an effect on SGs measured after 2 h of recovery (Supplementary Fig. 1f,g).

Thus, trehalose pretreatment primes cells for faster translational recovery and associated SG disassembly.

### Trehalose upregulates basal p-eIF2α levels and PP1 phosphatase subunits

**Figure 3:**
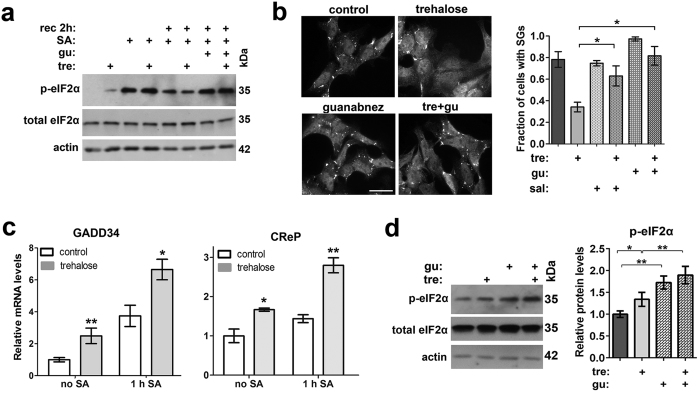
Trehalose causes upregulation of basal p-eIF2α and PP1 regulatory subunits CReP and GADD34. (**a**) Guanabenz inhibits p-eIF2α dephosphorylation during recovery from SA equally well in the presence and in the absence of trehalose. Cells were treated as indicated, with 100 mM trehalose added 1 h prior to stress or for 2 h to non-stressed cells; guanabenz (50 μM) was added immediately after washing off SA. A representative Western blot is shown. Full-size images of blots are available in the Supplementary Information. (**b**) Guanabenz and salubrinal partially reverse the effect of trehalose on SG clearance (n = 3, *p < 0.05). Cells were pretreated with 100 mM of trehalose for 1 h, stressed with SA and left to recover in the presence of 50 μM guanabenz or 40 μM salubrinal for 1.5 h. A representative image for each condition is also shown. Scale bar, 10 μm. (**c**) Trehalose induces upregulation of the stress-inducible (GADD34) and constitutively active (CReP) PP1 subunits at the mRNA level, and this effect persists after stress (n = 6, *p < 0.05; **p < 0.01). Cells were pretreated with 100 mM trehalose for 1 h. (**d**) Guanabenz and thehalose have an additive effect on p-eIF2α levels. Cells were exposed to trehalose (100 mM) and/or guanabenz (5 μM) for 24 h. A representative blot and quantification of band intensities are shown (n = 5, *p < 0.05, **p < 0.01). Full-size images of blots are available in the Supplementary Information.

We next wanted to understand why cells pretreated with trehalose are characterised by accelerated post-stress decrease in p-eIF2α levels. p-eIF2α is dephosphorylated by PP1 phosphatase that has two regulatory subunits, the constitutively expressed CReP and the stress-inducible GADD34. Dephosphorylation of p-eIF2α is known to be blocked by guanabenz, a selective inhibitor of GADD34^24^. Indeed, when added to cells immediately before recovery, guanabenz blocked dephosphorylation of p-eIF2α equally well in the presence and in the absence of trehalose (Fig. 3a) and delayed SG disassembly in trehalose-pretreated cells, eliminating the difference between these and control cells at least at the 1.5 h of recovery time point (Fig. 3b). Another small molecule, salubrinal, which inhibits both GADD34 and CReP^25^, had a similar effect (Fig. 3b). These observations suggest that trehalose primes cells for faster dephosphorylation of p-eIF2α, which in turn initiates restoration of translation and disassembly of SGs during the recovery phase.

Restoration of normal stress recovery pattern in the presence of inhibitors of PP1 phosphatase subunits hinted to the possibility that the observed effect of trehalose on p-eIF2α dephosphorylation during recovery from acute stress could be mediated via these subunits. Indeed, when compared to control cells, GADD34 and CReP expression were upregulated in cells treated with trehalose for 2 h (Fig. 3c, “no SA” bars) or pretreated with trehalose and stressed with SA (Fig. 3c“1 h SA” bars).

**Figure 4:**
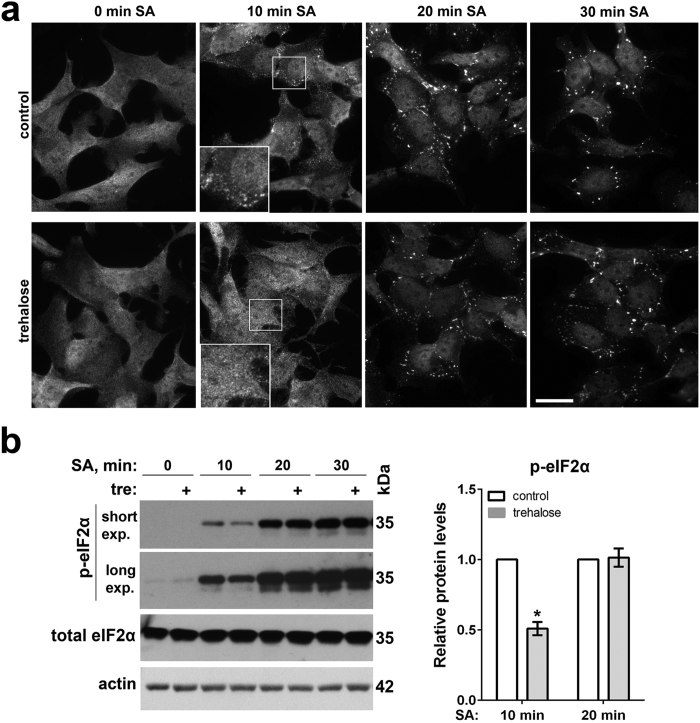
Trehalose delays SG assembly during stress. (**a**) Trehalose pretreatment changes the dynamics of SG assembly. Cells were pretreated with trehalose (100 mM for 1 h) or left untreated, and SA was added for 10, 20 or 30 min (representative images are shown). Scale bar, 10 μm. (**b**) Trehalose pretreatment results in reduced p-eIF2α levels during stress. Cells were treated with trehalose as in the panel a and harvested at the indicated time points. Representative blots and quantification of band intensities for p-eIF2α are shown (n = 4, *p < 0.05). Full-size images of blots are available in the Supplementary Information.

What can be behind upregulation of phosphatase subunits in trehalose-treated cells? We noticed that in unstressed cells treated with trehalose for 1 h the level of p-eIF2α was consistently elevated (Figs 2b, 3a and 4b). Similarly, a small but statistically significant and reproducible increase in the basal level of p-eIF2α was observed after prolonged trehalose treatment (24 h in Fig. 3d), indicating that trehalose might cause a mild stress. Guanabenz further increased the level of p-eIF2α when added to cells together with trehalose (Fig. 3d), indicating that phosphorylation of eIF2α in these cells is controlled, at least partially, by the availability of GADD34 phosphatase regulatory subunit. Therefore, mild stress response caused by trehalose is compensated to some extent by increased amount on phosphatase regulatory subunit(s) and consequent higher rate of p-eIF2α dephosphorylation.

Taken together our data suggested that basal increase in p-eIF2α levels in the presence of trehalose transcriptionally upregulates PP1 subunits and promotes p-eIF2α dephosphorylation after stress.

### Trehalose delays the build-up of p-eIF2α levels and SG assembly during oxidative stress

CReP and/or GADD34 are upregulated in the presence of trehalose before the application of strong acute stress. This should postpone the build-up of p-eIF2α levels during stress, which could result in a delay in SG assembly. We examined SG formation and p-eIF2α levels in trehalose-pretreated cells harvested 10, 20 and 30 min after stressing them with SA. SG assembly was visibly delayed after 10 min of SA exposure in trehalose-pretreated cells (Fig. 4a), and this correlated with downregulation of p-eIF2α at this time point (Fig. 4b). However this effect of trehalose pretreatment was transient - SG assembly and the build-up of p-eIF2α were indistinguishable in control and trehalose-treated cells already after 20 min of SA (Fig. 4a,b). Therefore, trehalose pretreatment can affect the dynamics of SG assembly during stress.

### Trehalose pretreatment affects basal translation

**Figure 5:**
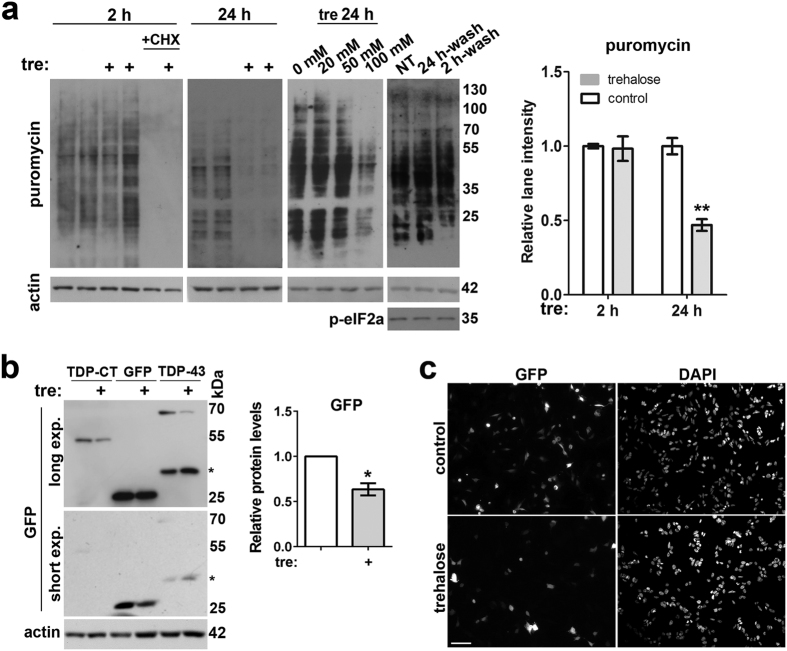
Trehalose decreases basal translation and expression of exogenous proteins. (**a**) Prolonged treatment with 100 mM trehalose impairs protein translation and this effect is reversible. A representative blot and quantification of lane intensities are shown (n = 4, **p < 0.01); note that presence of cycloheximide completely eliminates puromycin reactivity. For washing off trehalose, trehalose-containing media was replaced by normal media after 2 h (2 h-wash) or 24 h (24 h-wash) of trehalose exposure for additional 24 h before analysis. (**b,c**) Presence of trehalose in the media decreases levels of soluble and aggregate-prone transiently expressed proteins. Cells were transfected with plasmids to express GFP-tagged proteins full-length TDP-43, C-terminal TDP-43 fragment (TDP-CT) or GFP alone. Trehalose treatment started 6 h after addition of DNA-lipofectamine complexes and 16 h after beginning of the treatment cells were harvested for Western blotting and fixed for immunofluorescence. A representative blot is shown and band intensity for trehalose-treated cells relative to control cells (mean ± SEM) was quantified for GFP (n = 4, *p < 0.05) (**b**). Asterisk indicates a non-specific band. Full-size images of blots are available in the Supplementary Information. Representative images of control and trehalose-treated GFP-expressing cells are shown (**c**). Scale bar, 50 μm.

Increased basal p-eIF2α in trehalose-pretreated cells suggested that protein translation can be attenuated in these cells. Indeed, global translation was negatively affected after prolonged trehalose treatment; this effect was concentration-dependent and reversible (Fig. 5b). However, even long exposure to the highest concentration of trehalose (100 mM) caused only ~50% inhibition of translation and did not trigger all-out stress response with SG formation. Moreover, expression of ATF4 and CHOP, two major downstream stress effectors of p-eIF2α, was not changed (Supplementary Fig. 2). Inhibited basal translation in the presence of trehalose suggested that expression of transiently transfected proteins will be decreased. We measured levels of a transiently expressed soluble protein, GFP, as well as levels of full-length TDP-43 protein and its aggregate-prone C-terminal fragment tagged with GFP. Levels of all three proteins were decreased upon prolonged trehalose exposure (Fig. 5b,c).

Thus prolonged treatment with trehalose significantly affects translational rates compromising expression of exogenous proteins including soluble ones.

### Trehalose pretreatment decreases p-eIF2α level and cell survival following different types of stress

**Figure 6:**
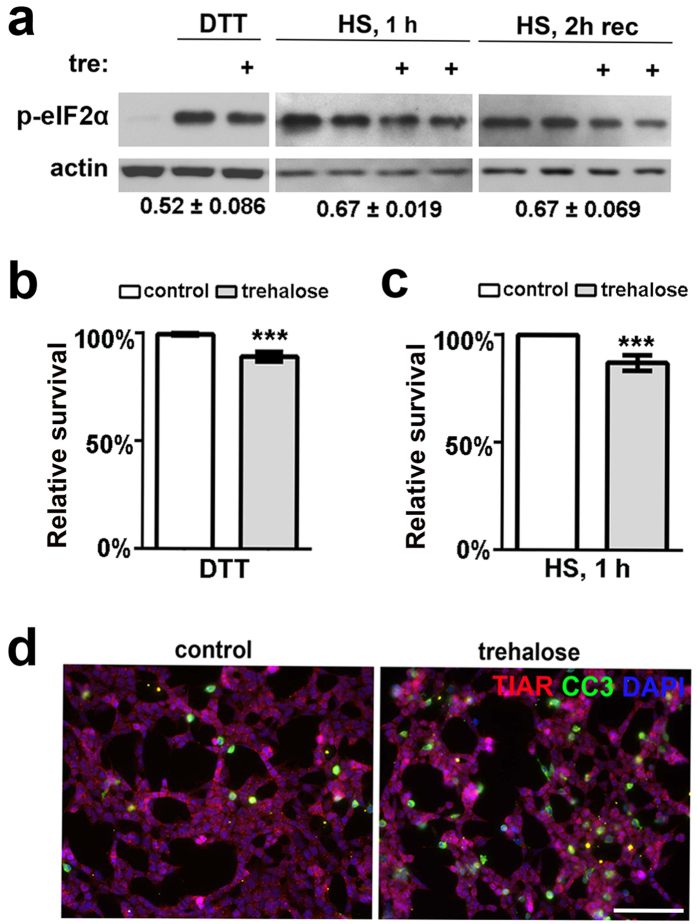
Trehalose pretreatment can sensitise cells to stress. (**a**) Trehalose pretreatment leads to early decrease in p-eIF2α levels in cells exposed to DTT or heat shock (HS). p-eIF2α levels were measured after 4 h of DTT exposure or immediately after HS (1 h) and after 2 h of recovery from HS. Representative Western blots and quantification of band intensities are shown (mean ± SEM, n = 4, p < 0.05). Full-size images of blots are available in the Supplementary Information. (**b,c**) Trehalose exacerbates the effect of DTT or heat shock. The bar chart shows survival of trehalose-pretreated (trehalose) and non-pretreated (control) DTT or HS-stressed cells measured using resazurin-based cell viability assay. The mean for non-pretreated cells was taken as 100% (n = 4, ***p < 0.001). (**d**) Trehalose promotes apoptosis during recovery from HS. Cells were co-stained with anti-TIAR and anti-cleaved caspase 3 antibodies. Representative images of both conditions are shown. Scale bar, 50 μm. In all experiments, cells were pretreated with trehalose (100 mM) for 1 h prior to application of a stressor.

To assess whether the decrease of p-eIF2α level in trehalose-pretreated cells is specific for SA-induced oxidative stress, we tested other types of acute stresses. Naïve cells or cells pretreated with 100 mM trehalose for 1 h were either treated with an ER stress inducer dithiothreitol (DTT, 1 mM) for 4 h or exposed to 43 °C heat shock (HS) for 1 h with consequent recovery at 37 °C. Trehalose-pretreated DTT-stressed cells displayed a decrease of p-eIF2α level compared non-pretreated cells; similarly, trehalose pretreatment led to decreased p-eIF2α levels after 1 h of HS and this effect persisted during the recovery period (Fig. 6a).

Accelerated recovery from stress has been reported to impair cellular viability^5^,^26^. We examined the effect of trehalose pretreatment on survival of cells exposed to DTT or HS. Whereas DTT itself did not significantly affect cellular viability, concomitant trehalose pretreatment reduced cell survival (Fig. 6b). HS resulted in decreased survival (71.1 ± 10.2% decrease from the pre-stress level after 1 h of HS, p < 0.001) and appearance of apoptotic cells positive for cleaved caspase 3; these effects were exacerbated by trehalose pretreatment (Fig. 6c,d).

## Discussion

The lifecycle and turnover of SGs have recently attracted close attention of researchers in the field of neurodegeneration. Malfunction of SG is believed to contribute to pathogenesis of many neurodegenerative conditions and normalisation of their function might be a promising therapeutic approach. Stimulation of autophagy was recently identified as one of means to promote SG disassembly^21^. However, it was not clear whether all autophagy-promoting compounds equally affect SGs. Moreover, there is still limited data on possible consequences of forced SG disassembly.

In present study we identified trehalose as a molecule potently promoting SG disassembly during recovery from a reversible oxidative stress. To our surprise, other autophagy-enhancing compounds did not affect SG disassembly rates, at least in the experimental setting we used. This is consistent with a recent finding that only a minor fraction of SGs is dependent on autophagy whereas most of them are dissolved via the activity of chaperone complexes^27^. Thus participation of autophagy in SG clearance is a complex phenomenon that likely depends on many factors, such as type of stress or SG composition.

Our data suggest that trehalose exerts its effect on SGs via the p-eIF2α pathway. By affecting p-eIF2α levels in basal and stressed state, trehalose modulates the ability of cells to maintain SG assembly following acute strong stress. The increase of basal p-eIF2α level by trehalose indicates that the treated cells undergo some sort of stress. However, this increase is not sufficient for triggering all-out tress response characterised by complete translational arrest and SG formation. It is feasible that elevated expression of PP1 phosphatase regulatory subunits GADD34 and CReP found in trehalose treated cells represents a mechanism of cell adaptation lessening the degree of cell stress response to mild stressors like trehalose. Surprisingly, induction of GADD34 by trehalose was not associated with induction of ATF4 or a pro-apoptotic factor CHOP, two proteins involved in transcriptional control of GADD34 levels under certain conditions^28^. This suggests the existence of an alternative mechanism of GADD34 regulation by elevated levels of p-eIF2α. We also examined whether CReP, a phosphatese subunit responsible for maintaining low basal p-eIF2α levels, might be behind the effect of trehalose on p-eIF2α levels using CReP knockout cells, however it does not appear to be the case (our unpublished observations). Thus, the increase in p-eIF2α levels in the presence of trehalose is likely to rely on the activity of a specific eIF2α kinase(s) and this needs to be addressed in future studies.

Although trehalose is widely used as an autophagy-promoting drug, its mechanism of action remains unknown. Since elevated p-eIF2α levels were reported to induce autophagy^29^,^30^, upregulation of basal p-eIF2α by trehalose may therefore be a mechanism behind its autophagy-stimulating properties.

We demonstrated that phosphorylation of eIF2α by trehalose is sufficient to significantly, although not dramatically, impair protein translation. Many studies of autophagic clearance of misfolded proteins relied on the use of trehalose at combination of concentration and time of exposure (100 mM for 24 h or longer) which severely impairs expression of transfected plasmids at the translational level resulting in the decrease of soluble protein and consequently of aggregated species. Thus it is hard to separate the effect of trehalose-mediated stimulation of autophagic protein clearance and the effect of attenuated translation of the exogenous protein in cells transiently overexpressing aggregate-prone proteins. This should be taken into consideration while designing such studies in the future.

Maintaining p-eIF2α levels above a certain threshold during stress was reported to be critical to ensure post-stress survival whereas early recovery decreases viability^5^,^26^ and we found the same pattern with trehalose. Therefore it should be kept in mind that approaches to force disassembly of “pathological” SGs may simultaneously affect normal, protective SGs which would be detrimental for cellular viability.

In conclusion, in present study we have identified previously unknown effects of trehalose on the p-eIF2α pathway, cellular translation and SGs. These data shall help better interpret previous results and design future studies of aggregate-prone proteins associated with neurodegenerative diseases with the use of trehalose in cell and animal models.

## Materials and Methods

### Cell maintenance and transfection

SH-SY5Y human neuroblastoma cells were maintained in Dulbecco’s modified Eagle’s medium, supplemented with 10% fetal bovine serum, 100x Penicillin/Streptomycin and 200 mM glutamine (all Invitrogen). Cells were transfected with pEGFP-C1 plasmid (Clontech) or plasmids encoding GFP-tagged, full-length WT TDP-43 and TDP-43 C-terminal fragment (aa 193–414) using Lipofectamine2000 according to manufacturer’s instructions. To obtain full-length and TDP-43 C-terminus, respective DNA fragments were PCR-amplified using cDNA from SH-SY5Y cells and AccuPrime polymerase and cloned into pEGFP-C1 vector.

### Treatments

#### Autophagy-enhancing compounds

Trehalose (Sigma): 10–100 mM; rapamycin (mTOR inhibitor; Calbiochem): 100 μM; LiCl (inositol monophosphatase inhibitor; Sigma): 10 mM; carbamazepine (inositol monophosphatase inhibitor; Sigma): 50 μM; verapamil and nifepidine (Ca2+ channel blockers; Sigma): 1 μM; amiodarone hydrochloride (mTOR inhibitor and Ca2+ channel blocker, Cayman): 10 μM; trifluoperazine hydrochloride (Ca2+ channel blocker, Cayman): 10 μM; clonidine hydrochloride (Gi-signaling activator; Cayman): 10 μM; Penitrem A (inhibitor of high conductance Ca2+-activated K+ channels; Cayman): 10 μM; fluphenazine (unknown; Cayman): 5 μM^22^,^31^,^32^,^33^,^34^,^35^.

#### Other chemicals

DTT (Sigma): 1 mM; sodium arsenite (Sigma): 0.5 mM; salubrinal (Santa Cruz): 40 μM; guanabenz (Sigma); bafilomycin A1 (Santa Cruz): 500 nM; chloroquine (Sigma): 10 μM.

### Cell toxicity assay

Cells were plated onto μClear™ 96-well black plates (Greiner Bio-One) at 80% confluency and treated as indicated in the Results section/figure legend. Cell viability was assessed using resazurin-based CellTiter-Blue^®^ Cell Viability Assay (Promega) according to manufacture instructions.

### Immunofluorescence on coverslips and SG analysis

Cells were prepared for fluorescent microscopy as described previously^36^. Briefly, cells were fixed with 4% paraformaldehyde on ice for 15 min, followed by washes with PBS and 5 min permeabilisation in cold methanol. After blocking in 5% goat serum/PBS/0.1% Triton X-100 for 1 h at room temperature, coverslips were incubated with primary antibodies diluted in blocking solution for 1 h at room temperature or at 4 °C overnight. Secondary fluorochrome conjugated antibody was added for 1 h at room temperature in dark place and nuclei were stained with DAPI. Coverslips were mounted on glass slides, on drop of Immumount mounting media (ThermoScientific). Fluorescent and phase contrast images were taken using BX61 microscope (Olympus), F-View II camera (Olympus) and Cell F software. Images were prepared using Photoshop CS3 or PowerPoint 2003 software. Cells possessing two or more large (mature) SGs (visualised by anti-TIAR staining) and total number of cells per a view field (x100 magnification) were counted in 20 or more randomly chosen fields (total ~200–300 cells per coverslip) blindly for the treatment, and mean ratio value was used for statistics. For analysis of correlation between p-eIF2a levels and SG numbers, p-eIF2α staining intensity in the cytoplasm of 30 cells was measured using ImageJ software and SGs were counted in the same cells. Correlation analysis was performed using GraphPad Prism software.

### RNA expression analysis

Total RNA from cells was extracted using RNAEasy mini-kit (Qiagen). First-strand cDNA was synthesised using random primers (Promega), and SuperScriptIII or SuperScriptII reverse transcriptase (Invitrogen). Quantitative real-time PCR was run in triplicate on an ABI StepOneTM real-time PCR instrument and data were analysed using StepOneTM Software v2.0 (Applied Biosystems) and the 2−ΔΔCT method with DyNAmo HS SYBR Green supermix and ROX (Finnzymes) as a passive reference dye. cDNA amount for each gene was normalised to that of GAPDH. Primer sequences used were as follows: GAPDH: 5′-TCGCCAGCCGAGCCA-3′ and 5′-GAGTTAAAAGCAGCCCTGGTG-3′; CHOP: 5′-TTAAAGATGAGCGGGTGGC-3′ and 5′-GCTTTCAGGTGTGGTGATGTA-3′; GADD34: 5′-GTAGCCTGATGGGGTGCTT-3′ and 5′-TGAGGCAGCCGGAGATAC-3′; CReP: 5′-TCGGTACAGCGTGACGTTC-3′ and 5′-TGGTCCTTTGCGATCCTCAT-3′.

### Puromycin labeling of newly synthesised proteins

Puromycin at a final concentration of 10 μg/ml was added directly to the media 30 min prior to lysis. In negative control samples, cycloheximide was added together with puromycin at a final concentration of 10 μg/ml. After several washes in 1xPBS, cells were lysed directly in SDS-PAGE loading buffer. Puromycilated proteins were detected by Western blotting using a monoclonal anti-puromycin antibody.

### Analysis of proteins by Western blotting

Total cell lysates were prepared by lysing cells on dishes in a loading buffer followed by denaturation at 100 °C for 5 min. SDS-PAGE and detection of proteins were carried out as described before^36^. Samples were normalised for equal loads of total eIF2α protein. Quantification of band or lane intensities of Western blots was performed using ImageJ software and mean intensity for control samples was taken as equal 1.

### Primary antibodies

Commercially available primary antibodies against the following antigens were used: eIF2α phosphorylated at Ser51: rabbit monoclonal, 119A11 (Cell Signaling) and rabbit monoclonal, ab32157 (Abcam); total eIF2α: rabbit monoclonal, D7D3 (Cell Signaling); LC3B: rabbit polyclonal, L7543 (Sigma); HSP70: mouse monoclonal, C92F3A-5 (Enzo Life Sciences); GFP: mouse monoclonal (sc-9996, Santa Cruz); ATF4: rabbit polyclonal, sc-200 (Santa Cruz); CHOP: mouse monoclonal, clone L63F7 (Cell Signaling); TIAR: mouse monoclonal, 610352 (BD Biosciences); cleaved caspase 3: rabbit polyclonal, 9661S (Cell Signaling); puromycin: mouse monoclonal, clone 12D10 (Merck Millipore); beta-actin: mouse monoclonal, A5441 (Sigma). Antibodies were used at 1:1000 dilution for all applications.

### Statistics

Non-parametric Mann-Whitney U-test or Kruskal-Wallis ANOVA with post-hoc Dunn’s test for multiple comparisons were used to assess significance of the difference between groups. All bar charts represent mean ± SEM, n corresponds to the number of biological replicates.

## Additional Information

**How to cite this article**: Dimasi, P. *et al*. Modulation of p-eIF2α cellular levels and stress granule assembly/disassembly by trehalose. *Sci. Rep.* **7**, 44088; doi: 10.1038/srep44088 (2017).

**Publisher's note:** Springer Nature remains neutral with regard to jurisdictional claims in published maps and institutional affiliations.

## Supplementary Material

Supplementary Figures

## References

[b1] Kedersha N. & Anderson P. Stress granules: sites of mRNA triage that regulate mRNA stability and translatability. Biochem Soc Trans 30, 963–969 (2002). 12440955 10.1042/bst0300963

[b2] Buchan J. R. & Parker R. Eukaryotic stress granules: the ins and outs of translation. Molecular cell 36, 932–941(2009). 20064460 10.1016/j.molcel.2009.11.020 PMC2813218

[b3] Kim W. J., Back S. H., Kim V., Ryu I. & Jang S. K. Sequestration of TRAF2 into stress granules interrupts tumor necrosis factor signaling under stress conditions. Mol Cell Biol 25, 2450–2462 (2005). 15743837 10.1128/MCB.25.6.2450-2462.2005 PMC1061607

[b4] Kedersha N., Ivanov P. & Anderson P. Stress granules and cell signaling: more than just a passing phase? Trends Biochem Sci 38, 494–506 (2013). 24029419 10.1016/j.tibs.2013.07.004 PMC3832949

[b5] Hofmann S., Cherkasova V., Bankhead P., Bukau B. & Stoecklin G. Translation suppression promotes stress granule formation and cell survival in response to cold shock. Mol Biol Cell 23, 3786–3800 (2012). 22875991 10.1091/mbc.E12-04-0296 PMC3459856

[b6] Arimoto K., Fukuda H., Imajoh-Ohmi S., Saito H. & Takekawa M. Formation of stress granules inhibits apoptosis by suppressing stress-responsive MAPK pathways. Nat Cell Biol 10, 1324–1332 (2008). 18836437 10.1038/ncb1791

[b7] Takahashi M. . Stress granules inhibit apoptosis by reducing reactive oxygen species production. Mol Cell Biol 33, 815–829 (2013). 23230274 10.1128/MCB.00763-12 PMC3571346

[b8] Thedieck K. . Inhibition of mTORC1 by astrin and stress granules prevents apoptosis in cancer cells. Cell 154, 859–874 (2013). 23953116 10.1016/j.cell.2013.07.031

[b9] Kedersha N. . Evidence that ternary complex (eIF2-GTP-tRNA(i)(Met))-deficient preinitiation complexes are core constituents of mammalian stress granules. Mol Biol Cell 13, 195–210 (2002). 11809833 10.1091/mbc.01-05-0221 PMC65082

[b10] Mazroui R. . Inhibition of ribosome recruitment induces stress granule formation independently of eukaryotic initiation factor 2alpha phosphorylation. Mol Biol Cell 17, 4212–4219 (2006). 16870703 10.1091/mbc.E06-04-0318 PMC1635342

[b11] Li Y. R., King O. D., Shorter J. & Gitler A. D. Stress granules as crucibles of ALS pathogenesis. J Cell Biol 201, 361–372 (2013). 23629963 10.1083/jcb.201302044 PMC3639398

[b12] Wolozin B. Regulated protein aggregation: stress granules and neurodegeneration. Mol Neurodegener 7, 56 (2012). 23164372 10.1186/1750-1326-7-56 PMC3519755

[b13] Ramaswami M., Taylor J. P. & Parker R. Altered ribostasis: RNA-protein granules in degenerative disorders. Cell 154, 727–736 (2013). 23953108 10.1016/j.cell.2013.07.038 PMC3811119

[b14] Aulas A. & Vande Velde C. Alterations in stress granule dynamics driven by TDP-43 and FUS: a link to pathological inclusions in ALS? Front Cell Neurosci 9, 423 (2015). 26557057 10.3389/fncel.2015.00423 PMC4615823

[b15] Figley M. D., Bieri G., Kolaitis R. M., Taylor J. P. & Gitler A. D. Profilin 1 Associates with Stress Granules and ALS-Linked Mutations Alter Stress Granule Dynamics. J Neurosci 34, 8083–8097 (2014). 24920614 10.1523/JNEUROSCI.0543-14.2014 PMC4051967

[b16] Kukharsky M. S. . Calcium-responsive transactivator (CREST) protein shares a set of structural and functional traits with other proteins associated with amyotrophic lateral sclerosis. Mol Neurodegener 10, 20 (2015). 25888396 10.1186/s13024-015-0014-y PMC4428507

[b17] Kim H. J. . Mutations in prion-like domains in hnRNPA2B1 and hnRNPA1 cause multisystem proteinopathy and ALS. Nature 495, 467–473 (2013). 23455423 10.1038/nature11922 PMC3756911

[b18] Shelkovnikova T. A., Robinson H. K., Southcombe J. A., Ninkina N. & Buchman V. L. Multistep process of FUS aggregation in the cell cytoplasm involves RNA-dependent and RNA-independent mechanisms. Hum Mol Genet 23, 5211–5226 (2014). 24842888 10.1093/hmg/ddu243 PMC4159159

[b19] Bentmann E., Haass C. & Dormann D. Stress granules in neurodegeneration–lessons learnt from TAR DNA binding protein of 43 kDa and fused in sarcoma. Febs J 280, 4348–4370 (2013). 23587065 10.1111/febs.12287

[b20] Protter D. S. & Parker R. Principles and Properties of Stress Granules. Trends Cell Biol 26, 668–679 (2016). 27289443 10.1016/j.tcb.2016.05.004 PMC4993645

[b21] Buchan J. R., Kolaitis R. M., Taylor J. P. & Parker R. Eukaryotic stress granules are cleared by autophagy and Cdc48/VCP function. Cell 153, 1461–1474 (2013). 23791177 10.1016/j.cell.2013.05.037 PMC3760148

[b22] Sarkar S., Davies J. E., Huang Z., Tunnacliffe A. & Rubinsztein D. C. Trehalose, a novel mTOR-independent autophagy enhancer, accelerates the clearance of mutant huntingtin and alpha-synuclein. J Biol Chem 282, 5641–5652 (2007). 17182613 10.1074/jbc.M609532200

[b23] Gilks N. . Stress granule assembly is mediated by prion-like aggregation of TIA-1. Mol Biol Cell 15, 5383–5398 (2004). 15371533 10.1091/mbc.E04-08-0715 PMC532018

[b24] Tsaytler P., Harding H. P., Ron D. & Bertolotti A. Selective inhibition of a regulatory subunit of protein phosphatase 1 restores proteostasis. Science 332, 91–94 (2011). 21385720 10.1126/science.1201396

[b25] Boyce M. . A selective inhibitor of eIF2alpha dephosphorylation protects cells from ER stress. Science 307, 935–939 (2005). 15705855 10.1126/science.1101902

[b26] Sidrauski C. . Pharmacological brake-release of mRNA translation enhances cognitive memory. Elife 2, e00498 (2013). 23741617 10.7554/eLife.00498 PMC3667625

[b27] Ganassi M. . A Surveillance Function of the HSPB8-BAG3-HSP70 Chaperone Complex Ensures Stress Granule Integrity and Dynamism. Mol Cell 63, 796–810 (2016). 27570075 10.1016/j.molcel.2016.07.021

[b28] Ma Y. & Hendershot L. M. Delineation of a negative feedback regulatory loop that controls protein translation during endoplasmic reticulum stress. J Biol Chem 278, 34864–34873 (2003). 12840028 10.1074/jbc.M301107200

[b29] Talloczy Z. . Regulation of starvation- and virus-induced autophagy by the eIF2alpha kinase signaling pathway. Proc Natl Acad Sci USA 99, 190–195 (2002). 11756670 10.1073/pnas.012485299 PMC117537

[b30] Kouroku Y. . ER stress (PERK/eIF2alpha phosphorylation) mediates the polyglutamine-induced LC3 conversion, an essential step for autophagy formation. Cell Death Differ 14, 230–239 (2007). 16794605 10.1038/sj.cdd.4401984

[b31] Balgi A. D. . Screen for chemical modulators of autophagy reveals novel therapeutic inhibitors of mTORC1 signaling. PloS Оne 4, e7124 (2009). 10.1371/journal.pone.0007124 PMC2742736 19771169

[b32] Sarkar S. . Lithium induces autophagy by inhibiting inositol monophosphatase. J Cell Biol 170, 1101–1111 (2005). 16186256 10.1083/jcb.200504035 PMC2171537

[b33] Williams A. . Novel targets for Huntington’s disease in an mTOR-independent autophagy pathway. Nat Chem Biol 4, 295–305 (2008). 18391949 10.1038/nchembio.79 PMC2635566

[b34] Renna M., Jimenez-Sanchez M., Sarkar S. & Rubinsztein D. C. Chemical inducers of autophagy that enhance the clearance of mutant proteins in neurodegenerative diseases. J Biol Chem 285, 11061–11067 (2010). 20147746 10.1074/jbc.R109.072181 PMC2856980

[b35] Barmada S. J. . Autophagy induction enhances TDP43 turnover and survival in neuronal ALS models. Nat Chem Biol 10, 677–685 (2014). 24974230 10.1038/nchembio.1563 PMC4106236

[b36] Shelkovnikova T. A., Robinson H. K., Connor-Robson N. & Buchman V. L. Recruitment into stress granules prevents irreversible aggregation of FUS protein mislocalized to the cytoplasm. Cell Cycle 12, 3194–3202 (2013). 24013423 10.4161/cc.26241 PMC3865015

