# Supplementary material for: Modulation of p-eIF2α cellular levels and stress granule assembly/disassembly by trehalose
Source: Sci Rep. 2017 Mar 9;7:44088. doi: 10.1038/srep44088 (PMC5343430; doi:10.1038/srep44088)
Supplement: Supplementary Figures [file srep44088-s1.doc]

**Modulation of p-eIF2α cellular levels and stress granule assembly/disassembly by trehalose**

Pasquale Dimasi^1^, Annamaria Quintiero^1^, Tatyana A. Shelkovnikova^1,2^*, Vladimir L. Buchman^1^*

**
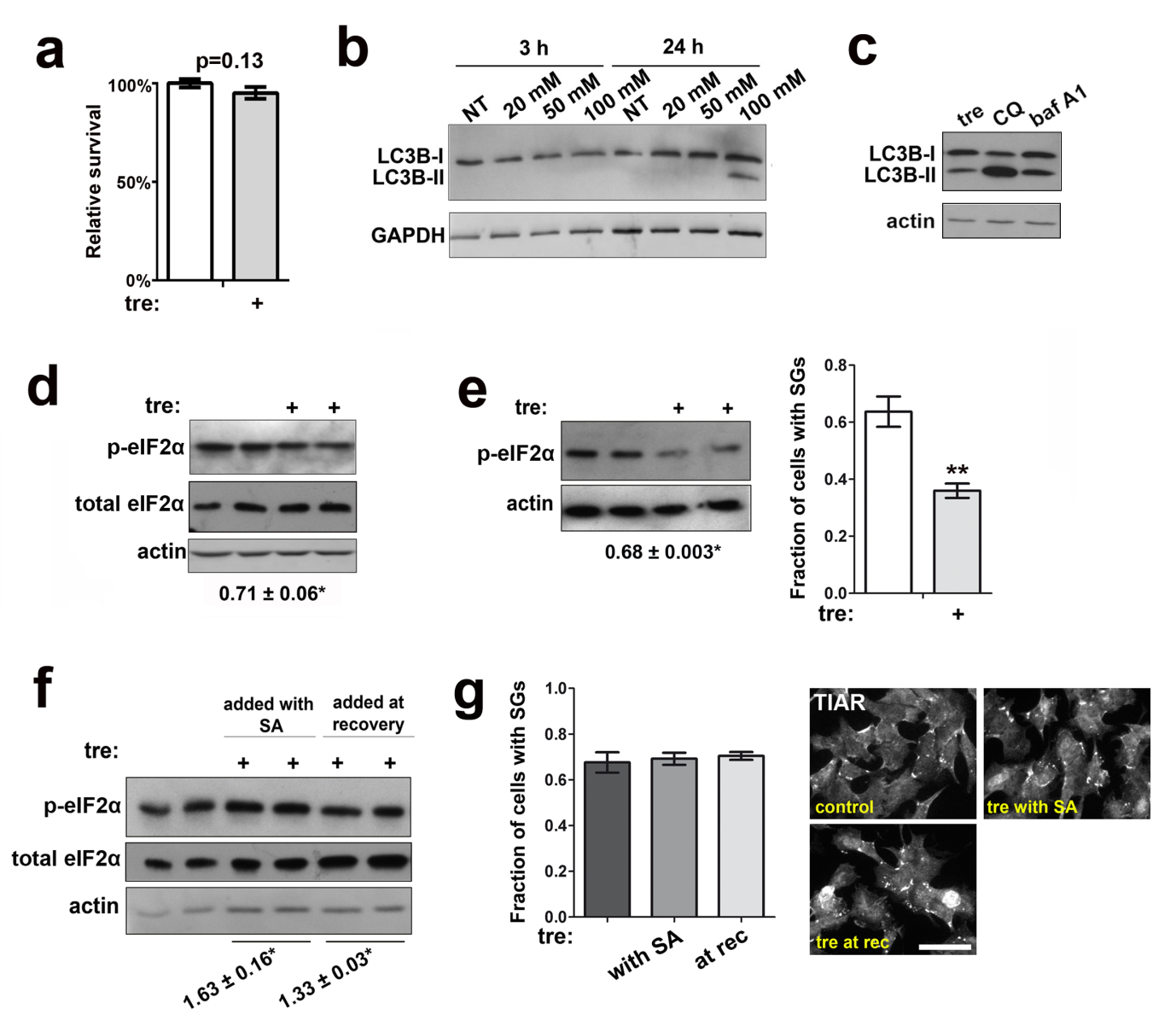
**

**Figure S1. Pretreatment is crucial for the effect of trehalose on p-eIF2α and SGs.**

**(a)** Trehalose does not exert significant toxicity even upon prolonged (24 h) treatment at high (100 mM) concentration. Cell survival was analysed using a resazurin-based assay.

**(b)** Only prolonged treatment with a high concentration of trehalose promotes LC3B-I to LC3B-II conversion.

**(c)** A representative Western blot demonstrating accumulation of LC3BII during bafilomycin A1 and chloroquine treatment. The cells were with trehalose (100 mM) for 24 h or autophagy inhibitors bafilomycin A1 (baf A1) or chloroquine (CQ) for 3 h.

**(d)** Prolonged trehalose treatment leads to reduced post-stress levels of p-eIF2α. Cells were pretreated with trehalose for 24 h prior SA stress and analysed after 1.5 h of recovery (n=3; *p<0.05).

**(e)** Pretreatment with trehalose, without its continued presence in the media during stress and recovery, also results in reduced post-stress p-eIF2α levels and accelerated SG dissolution. Cells were pretreated with trehalose for 1 h, at which point the media was replaced by trehalose-free media; immediately after, SA was added; cells were left to recover after SA stress in trehalose-free media for 2 h and harvested for Western blotting or SG counts. n=3; *p<0.05, **p<0.01.

**(f,g)** Lack of pretreatment cancels the effect of trehalose on p-eIF2α levels and SGs clearance. Trehalose added immediately before stress or before recovery leads to higher post-stress p-eIF2α levels (f, n=3; *p<0.05) and does not affect SGs (g). Trehalose was added to cells together with SA or immediately after washing off SA (at recovery); cells were left to recover after SA stress for 2 h and harvested for Western blotting or SG counts. Representative images of immunostained cells are shown. Scale bars, 10 µm.

**
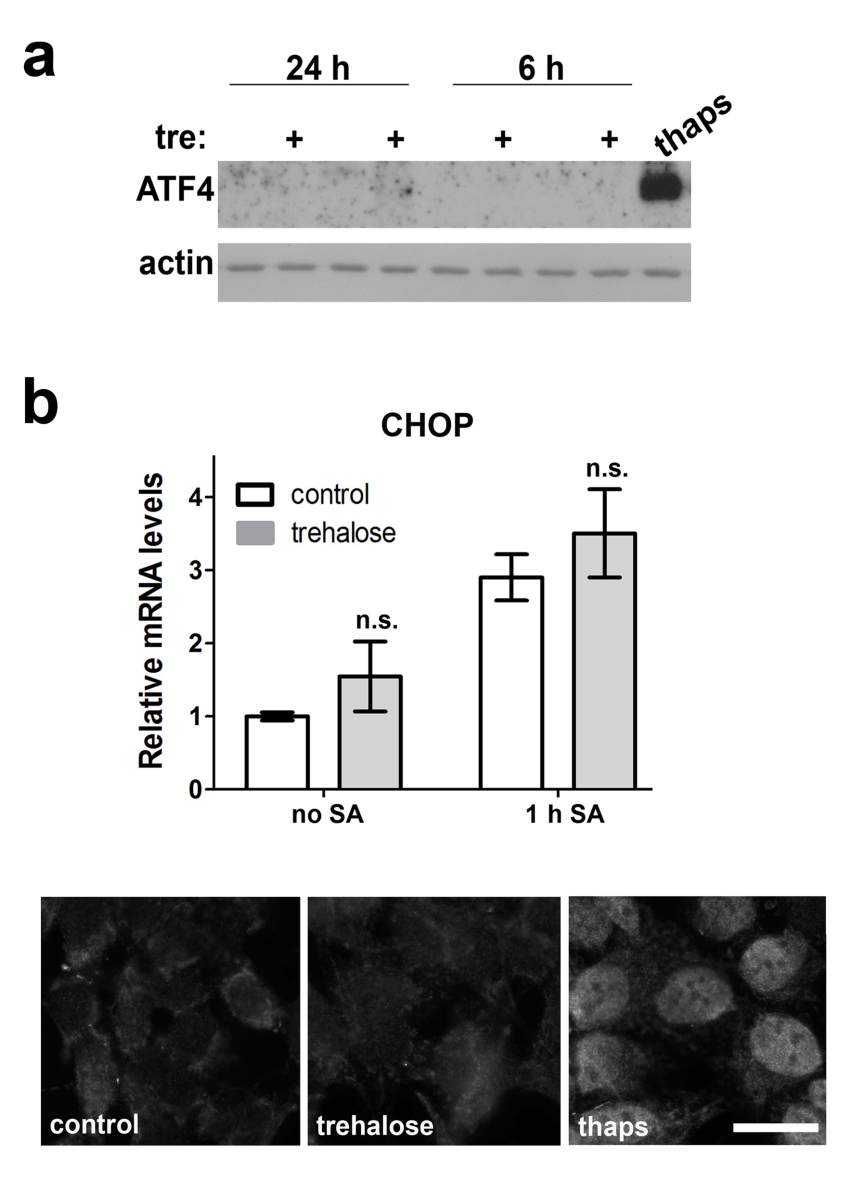
**

**Figure S2. Trehalose does not affect the expression of ATF4 or CHOP**

**(a)** Neither short nor long trehalose treatment induces ATF4 production in the absence of stress. Trehalose was added for the indicated times; thapsigargin was used as a positive control.

**(b)** Expression of an ATF4 target gene, CHOP, is not induced in the presence of trehalose. CHOP mRNA levels were measured by RT-qPCR (n=6) after 2 h of trehalose treatment with or without subsequent stress (SA). The presence of CHOP protein in the nucleus was determined by immunofluorescence, in this case thapsigargin was used as a positive control. Scale bar, 10 µm.

**Uncropped gel images**

**
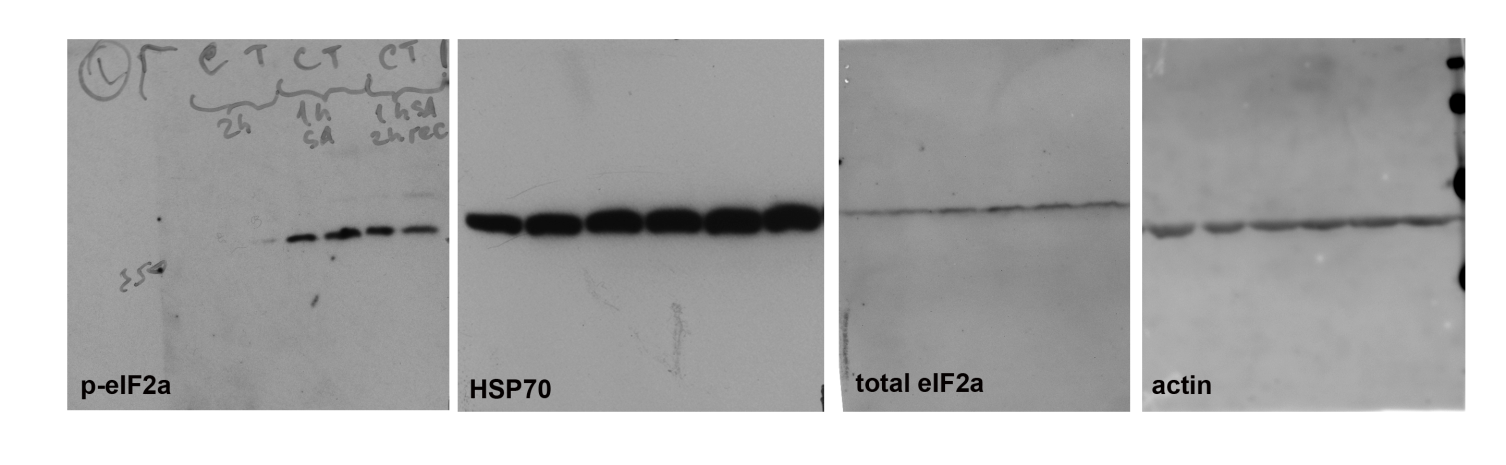
**

**Figure 2b**

**
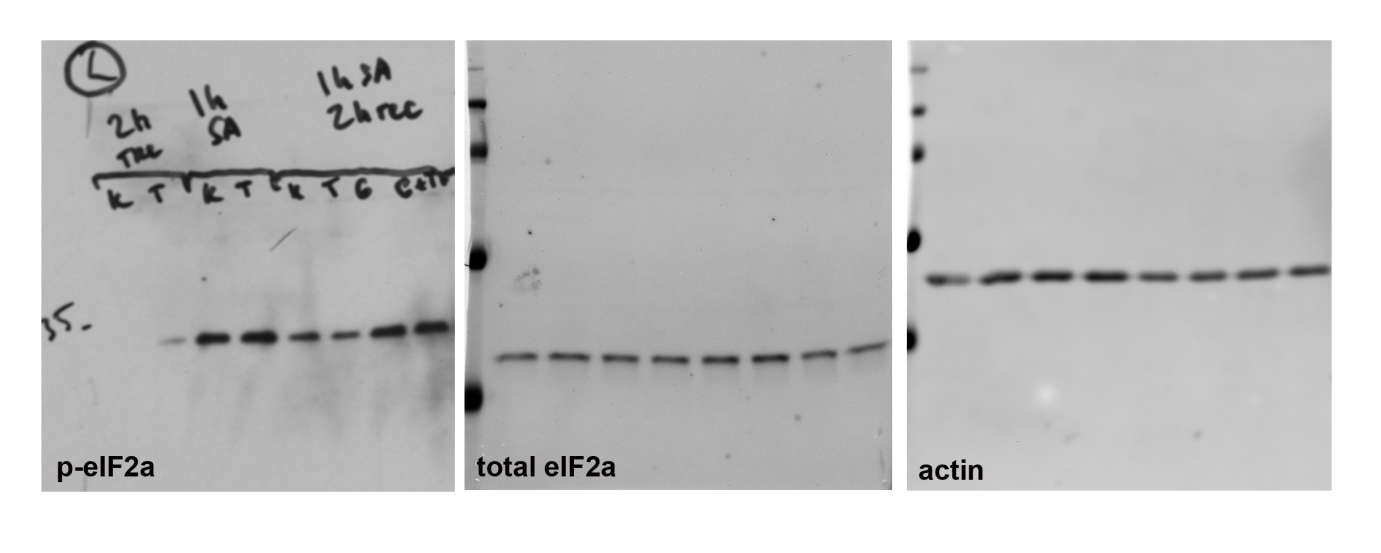
**

**Figure 3a**

**
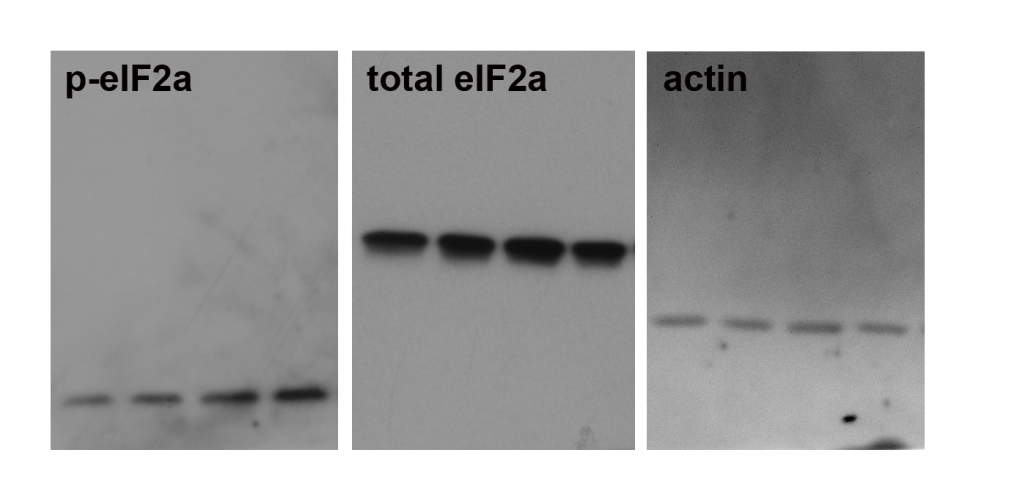
**

**Figure 3d**

**
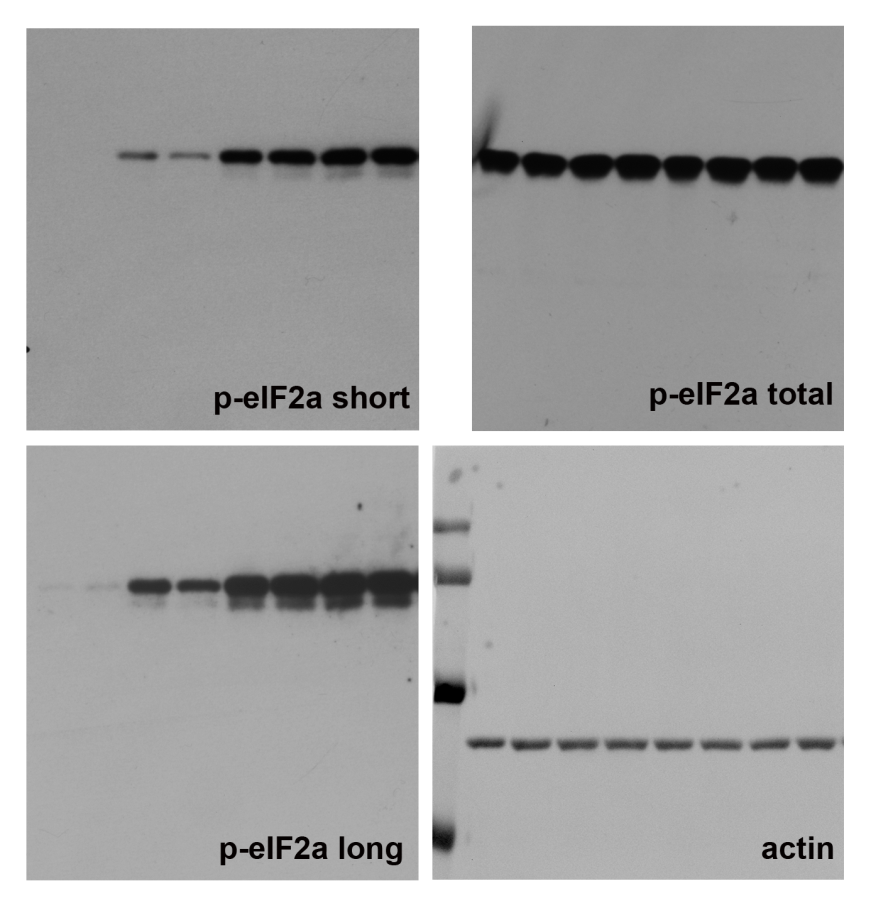
**

**Figure 4b**

**
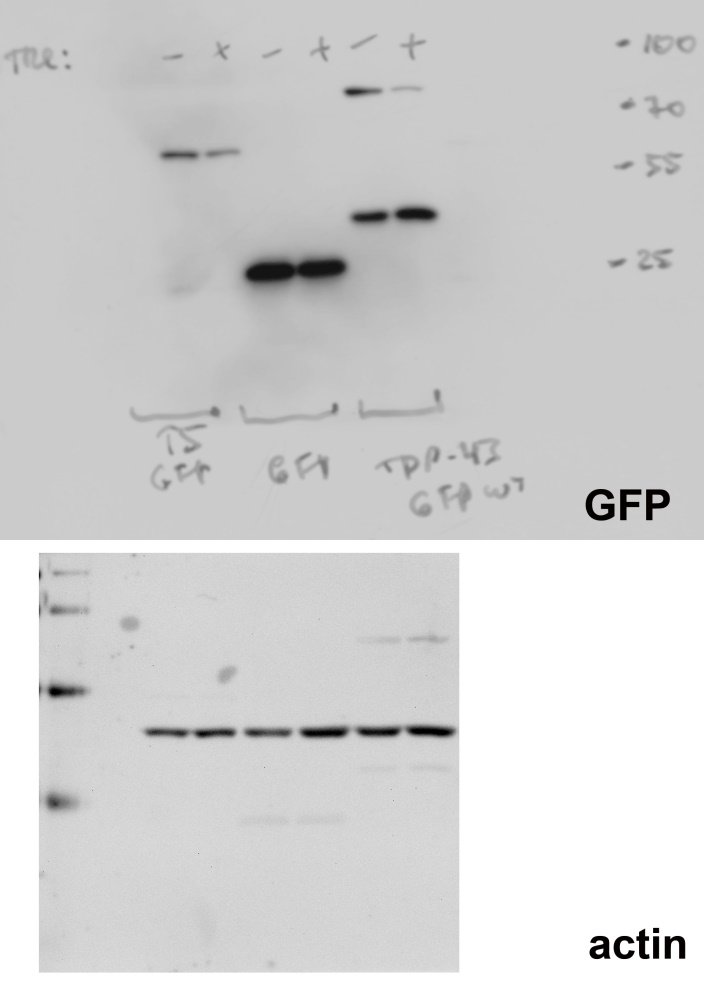
**

**Figure 5b**

**
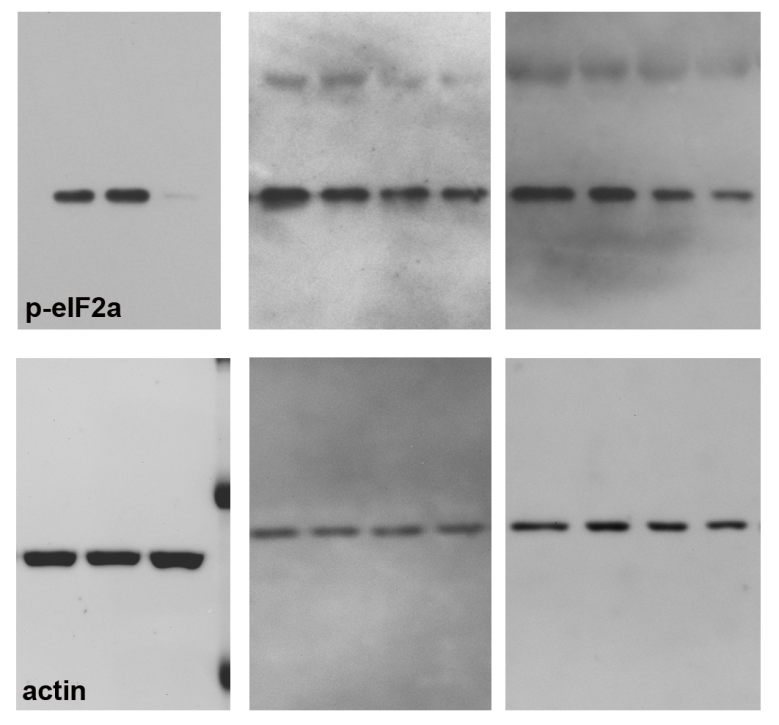
**

**Figure 6a**
